# Connect the dots: sketching out microbiome interactions through networking approaches

**DOI:** 10.20517/mrr.2023.25

**Published:** 2023-07-18

**Authors:** Marco Fabbrini, Daniel Scicchitano, Marco Candela, Silvia Turroni, Simone Rampelli

**Affiliations:** ^1^ Microbiomics Unit, Department of Medical and Surgical Sciences, University of Bologna, Bologna 40138, Italy.; ^2^ Unit of Microbiome Science and Biotechnology, Department of Pharmacy and Biotechnology, University of Bologna, Bologna 40126, Italy.; ^#^Authors contributed equally.

**Keywords:** Microbial networks, network interaction, network topology, ecological networks, community modeling, network approaches, shotgun metagenomics

## Abstract

Microbiome networking analysis has emerged as a powerful tool for studying the complex interactions among microorganisms in various ecological niches, including the human body and several environments. This analysis has been used extensively in both human and environmental studies, revealing key taxa and functional units peculiar to the ecosystem considered. In particular, it has been mainly used to investigate the effects of environmental stressors, such as pollution, climate change or therapies, on host-associated microbial communities and ecosystem function. In this review, we discuss the latest advances in microbiome networking analysis, including methods for constructing and analyzing microbiome networks, and provide a case study on how to use these tools. These analyses typically involve constructing a network that represents interactions among microbial taxa or functional units, such as genes or metabolic pathways. Such networks can be based on a variety of data sources, including 16S rRNA sequencing, metagenomic sequencing, and metabolomics data. Once constructed, these networks can be analyzed to identify key nodes or modules important for the stability and function of the microbiome. By providing insights into essential ecological features of microbial communities, microbiome networking analysis has the potential to transform our understanding of the microbial world and its impact on human health and the environment.

## NETWORK ANALYSIS

Immediately after the discovery of the microscopic world, it became clear that our world is dominated by, and extremely dependent on, a variety of complex microbial communities (or microbiomes, including bacteria, archaea, fungi, viruses, and protists). These communities play critical roles in several functions within different ecosystems (e.g., nitrogen and carbon cycling), but are also extremely important in the development and health maintenance of their associated hosts (e.g., plants and animal species)^[1-3]^. Indeed, these communities are not simply established by independent individuals, but all actors are strictly interconnected with each other and with the host so that they can communicate, cross-feed, recombine and coevolve^[4]^. For example, during the last decades, it has been seen that the human body serves as a host for several microbiomes, which also interact with human cells, among others playing a key role in the development of homeostasis and immune tolerance that are pivotal factors for health^[5,6]^. Referring to the human gut microbiota, thanks to numerous international projects such as the Human Microbiome Project, the American gut project, and MetaHIT, it has been widely recognized that it plays a critical role in human health and disease^[7,8]^. Indeed, an imbalance in the gut microbiota composition, generally referred to as dysbiosis, has been associated with a wide range of non-communicable diseases, for example, obesity^[9,10]^, diabetes^[11]^, inflammatory bowel disease^[12,13]^, and colorectal cancer^[14]^. More recently, other world programs, such as the Earth Microbiome Project, have extended the concept of human health to the entire planet, with a unified approach to optimizing the health of people, animals and the environment^[15]^. In the current context of global change linked to biodiversity loss, the goal of such initiatives is to characterize and map the planet microbiomes. This can help to understand their role, interactions, and importance in global change-related processes, not only for animal species but also for microorganisms^[16]^.

However, even though it is now clear that microbiomes play a central role in life and global health, we are just beginning to explore the variety of microbial communities and understand how these microbes interact with each other and with their hosts and environments^[4]^. Thanks to the advent of Next-Generation Sequencing (NGS) techniques, it has been possible to understand the high complexity of microbial communities and their layout associated with different conditions or pathologies. NGS techniques are extremely powerful for characterizing microbial communities through the sequencing of specific taxonomical molecular targets, such as the 16S rRNA gene for bacteria and the internal transcribed spacer of rRNA for fungi and specific bacterial species such as those belonging to *Bifidobacterium* or *Lactobacillu*^[17,18]^, or shotgun metagenomics for the evaluation of the composition and function of the whole community, including viruses. However, microbiome studies often encounter challenges related to isolation and sequencing biases. These issues can arise due to variations in sample collection, DNA extraction methods, amplification techniques, and sequencing platforms. It is critical to gain accurate insights from microbiome data. This requires rigorous quality control measures, normalization techniques, and the use of computational tools to correct for biases such as sequencing errors and taxonomic assignments.

Understanding the composition and functional potential of the microbiota and exploring the possible pairwise associations between a single taxon and a specific variable, condition or disease is just the surface of a deeper knowledge of how the structure and dynamics of a complex microbial community could shape human and planetary health^[16,19]^. Indeed, the intricate interplays that take place among microbial taxa and between them and their host emphasize the significance of the overall functions of the microbial community, often outweighing the importance of any function related to single taxa^[20,21]^. For this reason, a comprehensive understanding of the microbial community interactomes could enable researchers to better understand how microorganisms interact with each other and with a host, and how they are shaped by external perturbations^[19]^. Such understanding could lead to the design of novel and precise strategies in the clinical, agricultural, and bioremediation fields, just to mention a few, and, more widely, in the current climate change scenario, it could contribute to our better life on earth.

## NETWORK APPROACHES FOR MICROBIOME ANALYSIS

Considering all the aforementioned information, it becomes evident that understanding all microbiome interactions, whether related to health, disease, global change, or other conditions, is of paramount importance. A useful approach to better dissect these interactions and the high microbiome complexity in terms of compositional variability, dynamic nature of both structure and function, and ability to self-reproduce and self-organize is represented by network theory. In recent decades, various tools have been developed for different types of networks and are used nowadays in several applications by biologists, mathematicians, social scientists, and computer scientists exploring interactions between entities. For example, they have been applied in infectious disease research^[22]^, social interaction analysis applied to marketing^[23]^ and political science^[24]^, analysis of neuroimaging data^[25]^, information flow through the internet^[26]^, and genomics data analysis^[27]^. Recently, it has emerged that the microbiome also falls within the applicability of network theory because the architectural features of networks appear to be universal in any complex system^[4,28]^. This universality has made it possible to use tools and theories developed in well-studied non-biological systems to characterize the intricate relationships that define the high complexity of microbial interactions, such as mutualism, synergism, commensalism, or parasitism^[28]^. To date, several methods have been used in microbiome studies to construct ecological networks, ranging from simple pairwise Pearson or Spearman correlation measures to more complex multiple regression and Gaussian graphical models. The main differences between these methods are efficiency, accuracy, speed, and computational requirements. To ensure a comprehensive understanding, it is important to first describe the main components that make up a network before proceeding to explain these methods in detail.

**Figure 1:**
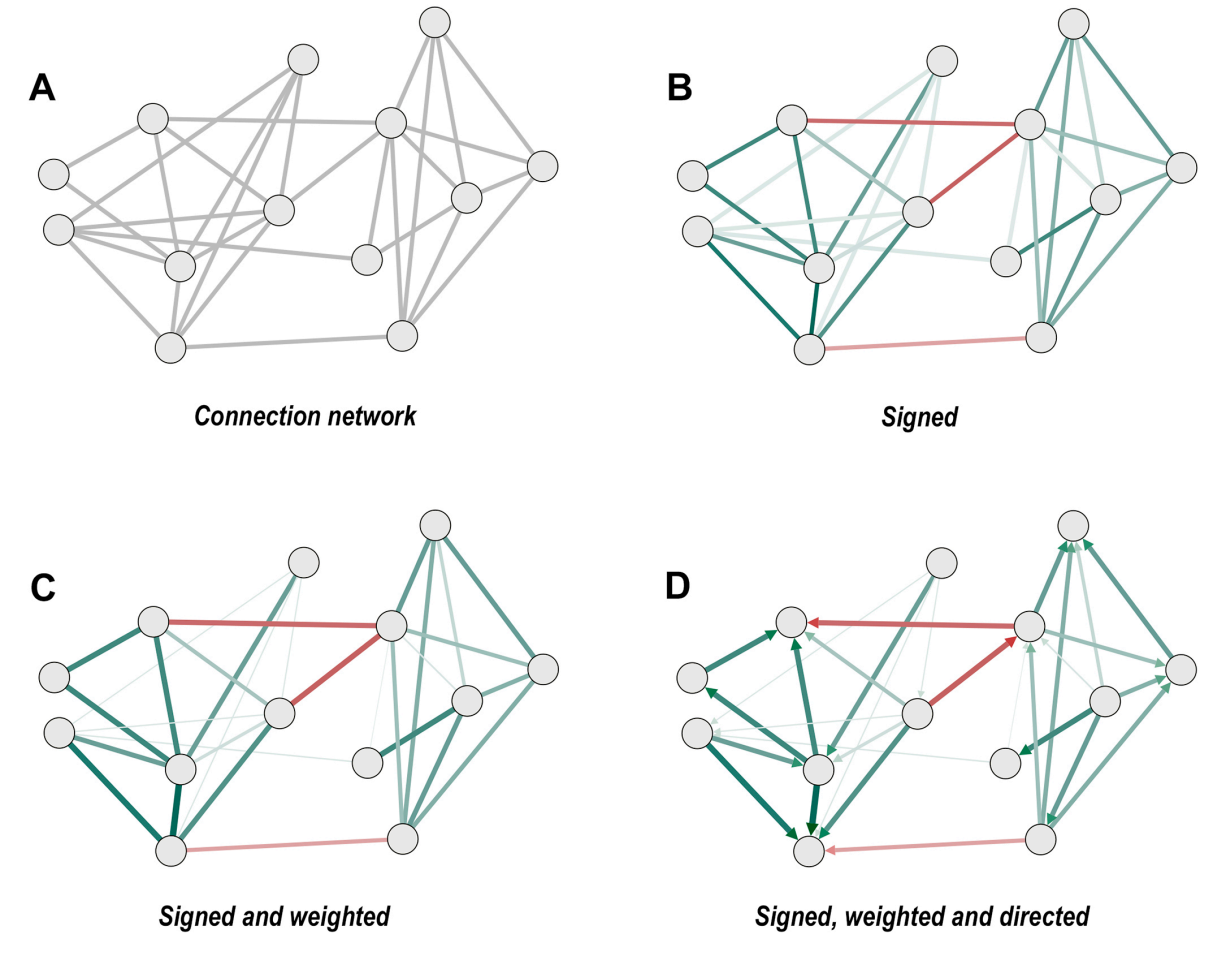
General structure of interaction networks. (A) A general network structure where nodes represent an entity (e.g., a taxon) and edges represent pairwise relationships. (B) The same network structure with signed relationships according to their positive (green) or negative (red) nature. (C) Network with both signed and weighted relationships, where the line thickness is proportional to relationship’s strength (derived either from adjacency, similarity, or correlation values). (D) Signed, weighted, and directed network, where relationships are represented by an arrow, to highlight the causal influence of a node on another one.

Networks, also called graphs, are defined as a set of mathematical concepts for describing and examining the relationships between system entities. Most biological systems can be described as networks where the nodes can be, for example, metabolites in a metabolic network, genes or regulators in a gene regulatory network, and microbial taxa in a microbiome network. The nodes represent the entities, and the edges are plotted to visually depict the interactions that connect these entities. These interactions can be either negative or positive, forming the graphical visualization of the relationships between the nodes. These ecological interactions between one microorganism and another can be described by both the weight and the sign of the interaction [Figure 1]. Based on the characteristics of the interactions, the networks are called “weighted”, if it is possible to quantify and represent the strength of the interaction, and “signed” if both positive and negative values are represented^[19]^. If the relationships are weighted, signed, and have a direction, they can be defined in terms of a source and a target, so the network is classified as “directed”. However, regarding microbiome analysis, generally, it is impossible to define the direction of the interaction, so we generally talk about an “undirected” network.

**Figure 2:**
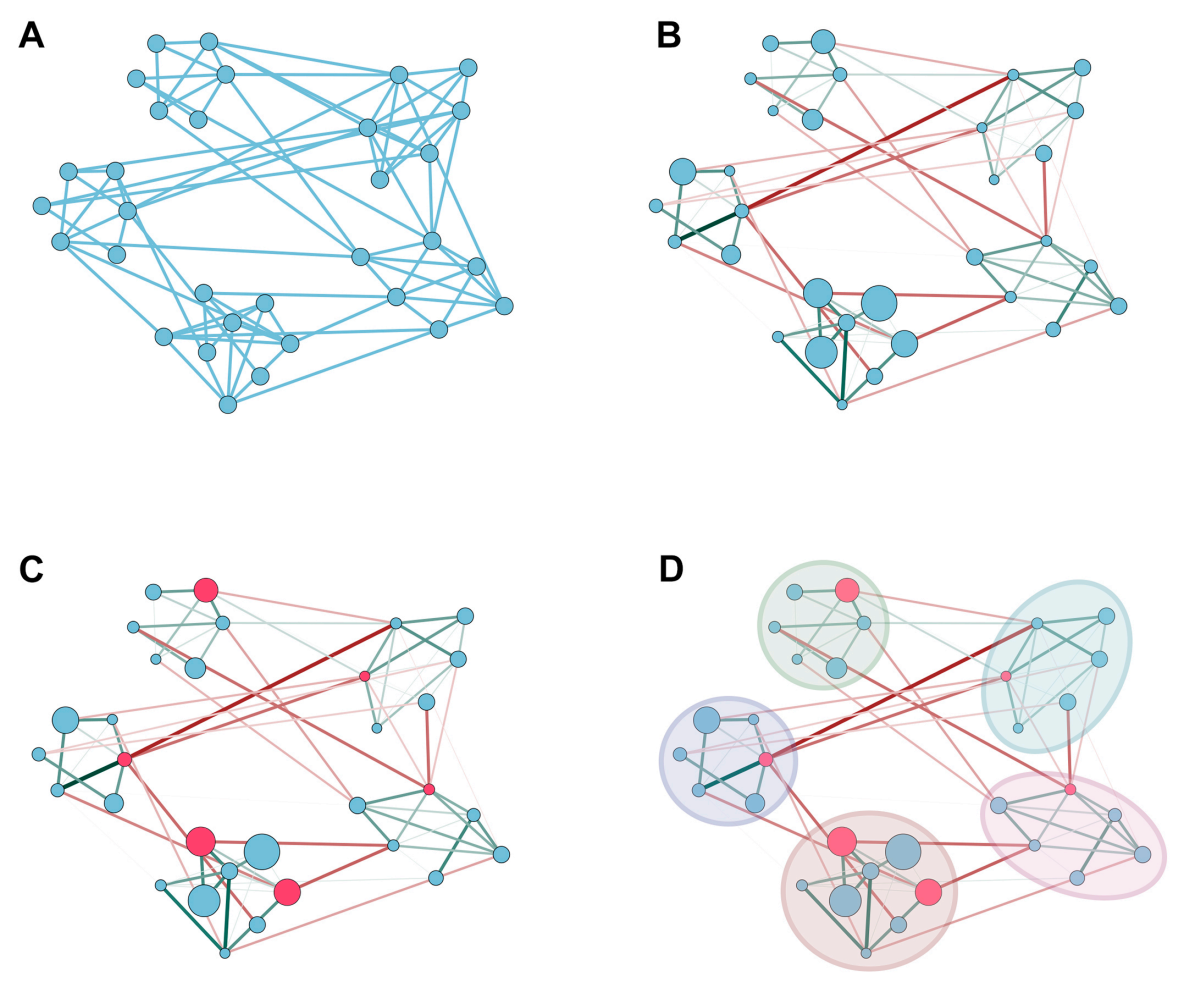
Concepts of networking applied to microbiome data. (A) Example of a network constructed from a relative abundance table of bacterial genera, where each node represents a bacterial genus within the microbial community and each blue line represents a pairwise relationship. (B) The same network structure, with green and red lines highlighting positive and negative interactions, respectively. Node size is proportional to the relative abundance of each bacterial genus and edge thickness to the force of relationships. (C) Microbiome network with hub nodes highlighted in red. (D) The microbiome network is grouped into five distinct modules, highlighted by different colored circles. The image was created using Cytoscape.

Once the networks have been built, based on the data and the different methods, there are various topological or ecological parameters useful for describing and analyzing the overall structure of the system. For example, there are three main metrics used to define node characteristics [ Box 1]: 1) “degree”, defined as the number of correlations of a node with the others; 2) “betweenness”, defined as the shortest path between each pair of nodes in the network; and 3) “closeness”, calculated as the reciprocal of the sum of the distances from a given node to all reachable nodes^[29]^. Based on these parameters, it is possible to define other topological properties of a network such as “hub nodes”, “keystone nodes” and “network modules”, all described in Box 1 as well and shown in Figure 2. Regarding network ecological parameters, several network properties have been widely used to predict network stability in a lot of field studies investigating, for example, plant-pollinator networks^[30]^ and food webs^[31]^, and have recently been applied in microbiome studies^[32-34]^. Properties such as modularity and the ratio of negative to positive interactions are some of the most important. The first, modularity, describes how strongly taxa are compartmentalized into modules. Generally, greater modularity is characteristic of a more stable community because losing taxa from a module prevents it from affecting the rest of the network^[31-43]^. The second property is also associated with community stability because a lower value of the ratio of negative to positive interactions highlights a higher presence of positive interactions, so in a microbiome network, when one member decreases in abundance, it might negatively impact the fitness of other associated taxa^[34,35]^. In general, communities with higher modularity and a higher ratio of negative/positive interactions are less sensitive to environmental perturbations and return more easily to an equilibrium state after a stressful condition^[33,35,36]^.

## TOOLS FOR NETWORKING MICROBIOME ANALYSIS

Before exploring networking approaches and the latest tools available, we must understand the nature of microbiome data and their weaknesses leading to the need for *ad hoc* procedures for network construction.

The microbiome data to date are primarily compositional, with 16S rRNA amplicon sequencing and shotgun metagenomics being the techniques of choice for profiling its arrangement. Microbial associations and interactions can thus be inferred based on the resulting abundance profiles via networking approaches. However, there are quite a few challenges in determining such associations among microbial taxa. First, the data are compositional and therefore subjected to variations in sampling and sequencing depth, with deeper samples more likely to have a higher fraction of assigned sequences. For this reason, relying on standard correlation methods such as Pearson or Spearman to evaluate the relationship between microbial proportions can lead to spurious associations^[37]^. In addition, microbiome data tend to be sparse, i.e., highly zero-inflated, and over-dispersed, as many taxa are likely to be observed in only a few samples. For this reason, assuming a standard distribution such as normal or Poisson on the compositional data may not be valid.

Table 1 lists the most common tools used for microbiome networking and described hereafter. Please use the "source" information in this table and refer to the developer's feature description for a clear and thorough explanation of those tools.

**Table 1:** List of the most common tools used for microbiome networking

**TOOL**	**METHOD**	**FORM**	**SOURCE**
*Correlation methods*			
SparCC	Log-transformed compositional Pearson correlation	-Python -R package	https://github.com/dlegor/SparCC.git
CCLasso	Log-ratio transformed abundances in latent variable model with L1-norm shrinkage method	R package	https://github.com/huayingfang/CCLasso.git
Canonical correlation and igraph network analysis	Correlation matrices (Pearson, Spearman), custom weighted coefficient thresholds and multiple testing correction	-Custom R codes -R package	https://igraph.org/
CoNet	Canonical correlation but with plenty of possible presets in a user-friendly interface	Cytoscape app	https://apps.cytoscape.org/apps/conet
*Graphical models*			
MInt	Poisson-multivariate normal hierarchical model	R package	https://rdrr.io/cran/MInt/
SPIEC-EASI	Neighborhood selection and sparse inverse covariance selection	R package	https://github.com/zdk123/SpiecEasi.git
SPRING	Semi-parametric rank-based correlation estimation with Meinshausen and Bühlmann neighborhood selection and stability-based approach for optimal tuning	R package	https://github.com/GraceYoon/SPRING.git
HARMONIES	Zero-inflated negative binomial distribution	-R package -Web tool	- https://github.com/shuangj00/HARMONIES.git - https://lce.biohpc.swmed.edu/harmonies/
FlashWeave	Local-to-global learning constraint-based causal inference framework	Julia CLI	https://github.com/meringlab/FlashWeave.jl.git
MDiNE	Bayesian graphical model fit with MCMC methods	R package	https://github.com/kevinmcgregor/mdine.git
NetCoMi	Wrapper of network construction tool with tailored corrections ad adjustments	R package	https://github.com/stefpeschel/NetCoMi.git
*Multi-omic data integration*			
DIABLO	Multi-omic integration via correlation and dimension reduction methods	R package	http://mixomics.org/mixdiablo/ https://doi.org/doi:10.18129/B9.bioc.mixOmics
MiBiOmics	Multi-omic integration via correlation and dimension reduction methods	-R package -Web tool	- https://gitlab.univ-nantes.fr/combi-ls2n/mibiomics - https://shiny-bird.univ-nantes.fr/app/Mibiomics

### Correlation methods

To cope with the aforementioned challenges, several approaches have been developed to estimate correlation or covariance matrices in case of compositional constraints. For example, SparCC^[38]^ estimates linear Pearson correlations, but considering log-transformed components, approximating the correlation coefficients assumes that the number of components is large, and that the correlation network is sparse. CCLasso^[39]^ has been developed to address the limitations of SparCC, namely the approximate assumptions and resulting accuracy. The tool makes use of log-ratio transformed abundances as well but implements a latent variable model with L1-norm shrinkage method (also known as ‘LASSO’). This solves the constant sum constraint problem, which refers to the requirement that the proportions or abundances of different components within a sample must sum up to a constant value (usually 1 or 100). In the L1-norm shrinkage method, the goal is to estimate the coefficients of a linear regression model while simultaneously performing variable selection by imposing a penalty term on the absolute values of the coefficients. This penalty term encourages some coefficients to shrink towards zero, effectively performing variable selection and reducing the impact of irrelevant variables, potentially overcoming the constant sum constraint problem and yielding meaningful results in the analysis of microbiome data. While CCLasso performs better than SparCC, it has similar difficulties common to all networking correlation methods, mainly the inability to detect nonlinear relationships among taxa.

Nonetheless, among the possibilities to tackle the problems inherent to microbiome data, custom multiple comparison adjustment and strict threshold might be applied to correlation approaches to derive correlation matrices with significant correlations, representative of the interactions between taxa, which can be used for network construction^[40]^. In addition, easy-to-use though less precise methods are available, such as the Cytoscape app CoNet^[41,42]^. The main strength of such an app is the possibility of computing a number of different correlations, similarities or dissimilarities, to score the association strength between taxa, all within one of the most used platforms for network visualization, along with esyN^[43]^.

These methods can be generally referred to as co-occurrence networking, where a network is constructed representing microbial variables (taxa) as nodes, and their co-occurrence or co-exclusion associative relationships as edges. Yet, this approach may miss causal relationships.

### Graphical models

Both correlation methods and graphical models are used to analyze the relationships between variables in a dataset, but they differ in approach and assumptions. Correlation methods assume that relationships between variables are linear and do not account for nonlinear relationships or other types of dependencies, while graphical models provide a way to represent conditional dependencies to obtain sparse networks reflecting direct relationships. Graphical models are typically constructed using probabilistic models such as Bayesian networks or Markov random fields, representing the probability distribution of the data and the relationships between variables as a graph, where the nodes depict the variables (in the microbiome field, taxa or functions) and the edges represent conditional dependencies between such variables. The use of probability theory to model the relationships between variables is one of the main advantages of graphical models, allowing for the estimation of causal relationships, including nonlinear relationships. To date, graphical models appear to be the best option for evaluating microbiome properties via networking approaches.

Tools implementing this logic to its simplest extent include: (i) MInt^[44]^, which implements a Poisson-multivariate normal hierarchical model to learn direct interactions from compositional data; (ii) SPIEC-EASI^[45]^, which relies on a two-step inference of the interaction graph from the transformed compositional data, namely neighborhood selection and sparse inverse covariance selection; (iii) SPRING^[46]^, which infers graphical models via semi-parametric rank-based correlation estimation with Meinshausen and Bühlmann neighborhood selection^[47]^ for the identification of sparse conditional dependencies from such estimated correlation and a final stability-based approach for optimal tuning of algorithm parameters; and (iv) HARMONIES^[48]^, implementing a zero-inflated negative binomial distribution to model the skewness and sparsity of microbiome data. All these tools address part of the issues related to the nature of microbiome data and provide reasonable, sparse, and interpretable networks.

Another tool worth mentioning is FlashWeave, which is based on the local-to-global learning (LGL) approach, a constraint-based causal inference framework for predicting direct relationships between variables. Due to the conservative handling of structural zeros, FlashWeave can show reduced statistical power, hampering smaller datasets. Yet, it exhibits a significant increase in both speed, by several orders of magnitude, and network quality compared to alternative methods, especially when dealing with heterogeneous sequencing data^[49]^.

In addition to the tools mentioned above, there are others that allow for the estimation of separate networks for groups defined by a binary variable, allowing the differences between each network to be recovered, while also providing interval estimates for each parameter and evaluating the impact of the covariates on the network properties. Examples of such tools are MDiNE^[50]^, which makes use of a Bayesian graphical model fit with Markov Chain Monte Carlo (MCMC) methods, and NetCoMi^[51]^, an all-around tool for single and differential network construction, analysis and comparison that encloses most of the aforementioned methods (e.g., Pearson correlation, Spearman correlation, SparCC, CCLasso, SPIEC-EASI, SPRING) as well as association and dissimilarity methods, combined in a modular and supervised fashion.

### Multiomics data integration

Networking analysis has thus emerged as a powerful approach for modeling microbiome data, oftentimes by integrating these data with other omics data to evaluate functional linkages. Microbiome multi-omics requires collecting multiple sorts of high-dimensional biological data, including those from amplicon (e.g., 16S rRNA) sequencing, shotgun metagenomics, metatranscriptomics, metabolomics, etc., from a microbiome sample and its environment or host. This kind of integration holds the potential to resolve functional mechanisms of the microbiome^[52]^; consequently, tools and methods have been produced to address these procedures.

Multi-omics integration mostly exploits correlation-based methods, such as the Patient Similarity Networks (PSN) and Weighted Gene Correlation Network Analysis (WGCNA)^[53]^, and dimension reduction methods such as Principal Component Analysis (PCA), Partial Least Squares regression (PLS) or Co-inertia Analysis (CIA). Dimension reduction techniques aim to reduce the high dimensionality of multi-omics datasets while preserving as much relevant information as possible. By reducing dimensionality, these methods facilitate the visualization, interpretation, and analysis of integrated multi-omics data. Canonical correlation analysis can identify linear relationships between multi-omics datasets by finding the canonical variates that maximize the correlation between datasets. It is often used to reveal shared biological signals across different omics layers. Network-based integration, on the other hand, combines multi-omics data by constructing and analyzing molecular networks. Network-based methods utilize graph theory and network analysis techniques to identify modules or communities of interconnected genes, proteins, or metabolites that are functionally related. Packages providing this type of analysis have been released and allow for easy implementation of such approaches. Examples include DIABLO^[54]^-part of MixOmics^[55]^-and MiBiOmics^[56]^, both available as R packages.

In recent years, machine learning-based integration has become increasingly relevant in data science, including multi-omics data integration. Machine learning algorithms, such as random forests, support vector machines, or deep learning models, can be used to integrate and analyze multi-omics data. These algorithms can capture complex relationships and patterns across multiple omics layers, enabling predictive modeling or classification tasks.

## ASPECTS TO CONSIDER WHEN CONSTRUCTING NETWORKS FROM MICROBIOME DATA

The first aspect to consider before starting a networking analysis using microbiome data is the sample size. Sample size in network analysis refers to the number of individual entities (*i.e.*, nodes representing variables or taxa) for which data are available. In network analysis, the sample size can be determined based on various considerations, including the number of samples that will be included in the smallest network, to ensure that even the smallest network computed in the study is going to be statistically robust and reliable. Determining *a priori* the minimum sample size required for co-occurrence microbiome networking analysis is difficult, as it depends on several factors, such as the complexity of the data and the strength of the relationships between the variables. In general, the larger the sample size, the higher the statistical power and the associated odds of detecting meaningful relationships between variables. Providing a specific number that is universally applicable for the minimum sample size in co-occurrence networking analysis is difficult, as it varies depending on the specific context and research objectives. However, some studies suggest that a sample size of 25-30 samples per group may be considered reasonable for such kind of analysis^[45-57]^. It is important to note that the quality of the data, the accuracy of the sequencing technology used, and the statistical methods used to infer the network can also influence the minimum sample size required for a robust analysis.

The second aspect to consider is the structure of the dataset, including the choice of using compositional (taxonomic) and/or functional data. Generally, in both cases, the data are present as tabular outputs reporting for each sample a given value (either relative abundances or counts, or normalized counts) for *n* observed features (e.g., taxa, pathways, etc.). Concerning compositional information, data can be obtained from: (i) 16S rRNA amplicon sequencing, often followed by QIIME 2^[58]^ bioinformatic pipeline processing; or (ii) shotgun metagenomics sequencing, followed by read alignment tools such as MetaPhlAn 4^[59]^, Kraken2^[60]^ and METAnnotatorX2^[61]^, to ultimately produce the compositional table. From the functional standpoint, inferred techniques starting from 16S rRNA data such as PICRUSt2^[62]^ can be used, yet shotgun metagenomics is highly preferred. The reason for this is that 16S rRNA amplicon sequencing methods rely on the use of reference sequences to analyze small amplicons derived from metagenomes, rather than examining the entire metagenome as a whole. Such a limitation might result in improper assessment of metabolic capability and inadequate taxonomic assignment to resolve microbiome compositional data down to the species level. The possibilities are vast regarding tools for functional annotation of metagenomic samples and include both read-mapping and assembly approaches. For what concerns read-mapping, the most commonly used tools include HUMANn3^[63]^, MetaCV^[64]^, EggNOG^[65]^, and other methods comprising the use of Hidden Markov Models on tailored databases. On the other hand, the use of assembly approaches includes some tools for species-level genome bin definition, such as MetaWRAP^[66]^, and some tools for functional annotation, such as Prokka^[67]^ and EggNOG^[65,68]^. The yield of metagenomics approaches often involves multiple layers of information such as taxonomic composition and functional profile, which require multi-omic integration to properly address their relationships. Multi-omics integration is arguably the most complex scenario and, probably because of this, receives the least coverage to date.

When considering the structure of the dataset, another important aspect is the decision of whether or not to filter the data. Including all variables, even low-abundance ones, may provide a more comprehensive view of the relationships between microbial taxa or functions detected, possibly revealing previously unknown associations. Nevertheless, this may also lead to increased network complexity (with increased computational resources and runtime requirements) and might result in weaker or spurious associations that could fade out the real relationships. On the other hand, including only the most abundant variables can simplify the network, possibly highlighting the most prominent relationships between microbial taxa or functionalities. This approach is particularly useful for studying the composition or function of a “core” microbiome, focusing attention on relevant microbial taxa and functional pathways while limiting the computational load. Accordingly, the choice between including all variables or considering only the most abundant ones (e.g., taxa/functions present only in the majority of samples, filtering out zero-values) should be based on the research question and the availability of computational resources, also taking into account the related limitations^[69-71]^. Typically, filtering procedures reduce the complexity of microbiome data while providing more reproducible and comparable results in microbiome data analysis. However, studies tend to use in-house arbitrary thresholds and no comparative studies on this topic are available to date. Given that the choice may affect the biological interpretation of the results, proceeding in both ways (*i.e.*, including everything and filtering something out) is not to be excluded. In this regard, providing the deepest and most accurate data possible is crucial for obtaining sound results. Consequently, deep shotgun metagenomics is generally preferred over 16S rRNA amplicon sequencing for the generation of compositional data for networking analysis.

Once eventually obtained tables of filtered data representing compositional or functional aspects of the microbiome, this can generally be fed directly to the previously reported tools to retrieve the edge list table, representing the pairwise connection between all nodes (microbial variables) with a corresponding strength for the connection computed according to the model chosen.

## CASE STUDY

**Figure 3:**
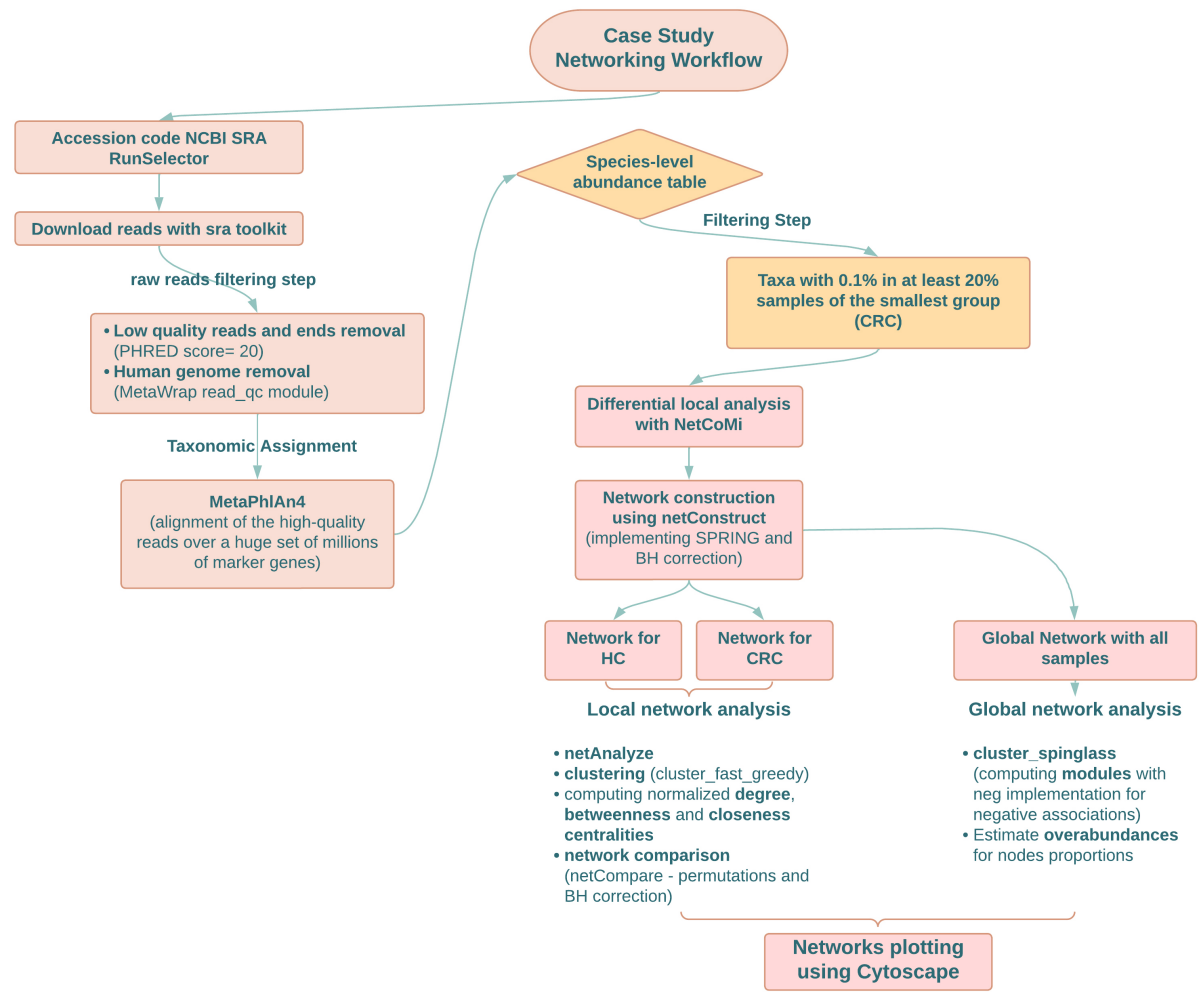
Flowchart of the pipeline used in the case study. General workflow of the case study, with a specific focus on the approaches and tools used for reconstructing and plotting microbiome networks. BH: Benjamini-Hochberg; CRC: colorectal cancer patients; HC: healthy controls. Created in Lucidchart, www.lucidchart.com.

In order to highlight the potential of networking analysis with microbiome data, we gathered shotgun metagenomic data from a recent work by Thomas *et al.* on a total of 201 subjects divided into 85 colorectal cancer (CRC) patients and 116 age-matched healthy controls (HC)^[72]^, with negative colonoscopy and no relevant gastrointestinal disorders [Supplementary Data 1]. The choice of this work is based on the high sequencing depth of the raw sequences produced by the authors and the simple clustering of the samples in the dataset (*i.e.*, CRC *vs.* HC). We decided to subsample the cohorts documented by Thomas *et al.*^[72]^, using a group sample size that is likely to be the most used by other research groups at this time. Quality- and human-filtered sequences showed an average depth of 9.94 Gb (± 0.31 SEM). We decided to limit the focus of this case study to species-level compositional networks in order to provide an example of a simple procedure, as detailed in Figure 3. The proposed procedure could be exploited by less experienced readers as well, without the need for complex and resource-demanding functional annotation pipelines. Sequences were processed via MetaPhlAn 4^[59]^ allowing for unclassified estimation, using the latest database available (vJan21-202103), and the analysis required around 3 TB of storage, max 100 GB of RAM and less than 3 days using 20 threads on an Intel Xeon Platinum 8260 Processor server. Compositional tables were then merged and processed through a coupled local-to-global networking analysis using NetCoMi with the SPRING method and adaptive Benjamini-Hochberg method for multiple test adjustments. The local networking approach consists in computing a separate network for each study group, so that pairwise network comparison techniques can then be used; the global networking approach, on the other hand, requires the construction of a single inferred network considering all the samples together and allows for the reconstruction of all the possible interactions in the dataset. The focus, in this case, is on identifying interaction modules and evaluating how such modules are populated by each group in terms of the overabundance of microbial components.

In order to compare the results obtained from standard approaches focusing on relative abundance and networking workflows, we first investigated the significant differences in relative abundance at the species level. To do so, Wilcoxon rank sum tests with false discovery rate (FDR) multiple comparison correction were used, and the results confirmed what was previously reported by Thomas *et al.*^[72]^: CRC patients showed higher relative abundances of the species *Clostridium symbiosum*, *Fusobacterium nucleatum*, *Gemella morbillorum*, *Peptostreptococcus stomatis*, *Porphyromonas somerae*, *Prevotella intermedia*, and *Parvimonas micra*, while for HC we detected higher proportions of *Roseburia intestinalis* [Supplementary Figure 1]. Given the subsampling of the original cohorts^[69]^, the concordance of the compositional findings with the original study is considered satisfactory.

To deepen the knowledge of the relationship between community members, we decided to reduce the size of the dataset by filtering out low-abundance species, in order to reduce the number of nodes to be computed in the network. To reduce the complexity of the dataset, we excluded the species that were detected with low relative abundances in only a few samples, setting arbitrary thresholds. Specifically, we retained only the species showing at least 0.1% relative abundance in at least 20% of the samples from the smallest group (in this case, CRC). We then conducted a local differential networking analysis with NetCoMi [Table 2], computing both edge and vertex connectivity. We detected a reduction in network modularity in CRC patients (log fold change = -0.317) and a slight increase in positive edge percentage (log fold change = 0.143). Other parameters that could be evaluated but showed no significant differences in our case included the clustering coefficient, relative network size, edge density, average path length, and natural connectivity. The differential analysis allowed us to evaluate the difference in terms of central nodes between the two networks, according to the degree, betweenness centrality, closeness centrality, and eigenvector centrality, ultimately leading to the identification of hub taxa. For example, comparing the two networks, we found significant differences in the Jaccard index (*P* = 0.032, Jacc = 0.190) in degree and a trend (*P* = 0.075, Jacc = 0.231) in betweenness and closeness centralities. Considering the normalized centrality measures, we classified a node as “hub” if it exhibited a difference of at least 0.3 (meaning a 30% difference in centrality compared to the other group). We scored *Clostridium fessum, Clostridium sp. AM22_11AC* and *Phocaeicola dorei* as hub taxa in the CRC group, and *Prevotella copri* clade B, *Prevotella copri* clade *C* and *Blautia caecimuris* in the HC one. We also computed connectedness and cohesion values for the networks according to Herren *et al.* to evaluate the ratio of negative to positive cohesion and total cohesion^[33]^. There was a notable decrease in both values in CRC (log fold change = -1.16 and -2.05, respectively).

**Table 2:** Topological and ecological parameters computed for the local networks of the case study

**PARAMETER**	**HC**	**CRC**	**LFC**
Modularity	0.416	0.334	-0.317
Positive edge percentage	73.694	81.365	0.143
NP ratio	0.121	0.054	-1.16
Total cohesion	0.306	0.074	-2.05

Modularity and positive edge percentage of the local networks were derived from the NetCoMi *netCompare* function, while the ratio of negative to positive cohesion and total cohesion was calculated as proposed by Herren *et al.*^[33]^. CRC: Colorectal cancer patients; HC: healthy controls; LFC: log fold change on base 2 logarithm; NP ratio: negative to positive cohesion ratio.

**Figure 4:**
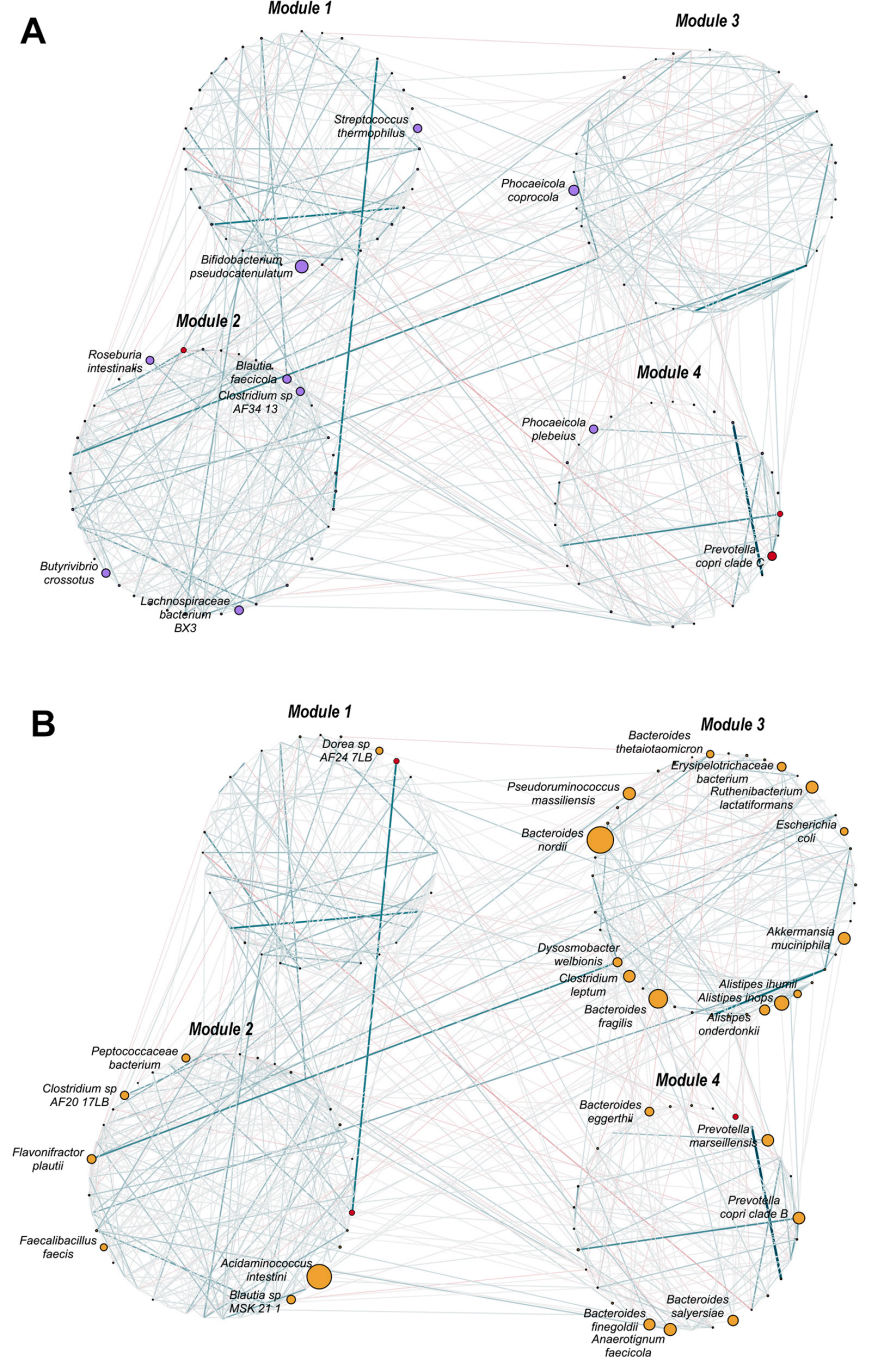
Global overabundance networks reconstructed from the case study. Networks for (A) healthy controls and (B) patients with colorectal cancer (CRC). Nodes and labels were plotted for nodes showing an overabundance greater than 1.35 ( + 35% average relative abundance in that group compared to the overall average relative abundance of that node in the dataset; represented in purple for HCs and yellow for CRCs in the plot). Node size is proportional to the overabundance value. Nodes colored in red represent the detected hubs by means of topological analyses of the global network, considering degree, betweenness, closeness and eigenvector centralities. The network layout was driven by the module detection with a mechanical spin-glass algorithm from the igraph R package, considering both positive and negative interactions. Such interactions between nodes are displayed as edges and colored according to the type of association (blue for positive interactions, red for negative ones), and line thickness is proportional to the adjacency values inferred during the network computation step. The figure highlights the relevance of networking as the CRC group clearly shows a different way of populating modules, with possibly harmful microorganisms particularly encompassed in the same module (*i.e.*, module 3), which appears to be characteristic of this group. Network images were created using Cytoscape. CRC: Patients with colorectal cancer; HC: healthy control.

When inferring the global network [Figure 4], we were able to determine the presence of four distinct modules according to a mechanical spin-glass algorithm, considering both negative and positive interactions, by processing the network edge list table produced by NetCoMi with the igraph R package. We populated the network by setting node sizes proportional to the overabundance values of each node in the given group, computed as the average relative abundance in that group divided by the overall average relative abundance of that node in the dataset. Nodes and labels were displayed for nodes showing at least 1.35 overabundance, meaning that a node was fully shown if the average abundance value in such group was at least 35% higher than the average relative abundance value of that taxon in the dataset. Edges were represented with thickness proportional to the adjacency values computed by SPRING, while the stroke color was established according to the association values of the computed matrices, given that the values ranged from negative to positive (-1,1). At first glance, it appeared clear that the HC group [Figure 4A] had an even distribution of relative abundances across nodes, with few of them highlighted in the overabundance network, and mostly included in modules 1, 2 and 4. On the other hand, the CRC group [Figure 4B] overpopulated the modules differently, especially enriching module 3.

## DISCUSSION

The topological differences detected in the case study using the local networking approach allowed us to detect a reduction in modularity in the CRC group, likely determined by the increase in positive edge percentage, which means that the nodes were more strongly interconnected among each other. This would result in a more probable spread of an external stressor towards the entire ecosystem, with possibly harmful consequences for the host. A more modular microbiome should instead be able to isolate the effect of external stressors on the microbial members of the modules most affected by the stressor, limiting the impact on the other members of the community. The negative to positive cohesion ratio confirmed these topological results showing a decrease in CRC, meaning that the community increasingly relies on positive interactions, as the stress induced by the cancer condition has probably leveled out the chances of establishing a variety of negative interactions in the microbiome. The reduction in total cohesion instead suggests an overall weakening of the forces that hold the community together, making the ecosystem even more fragile and exposing it to further stress. The detected hub nodes include taxa that were not reported as significant by compositional analyses and, with further studies, might provide additional insights to possibly help explain the biological mechanisms underlying CRC pathogenesis and progression. The global networking approach allowed for retrieving a comprehensive overview of the network structures, detecting the modules of closely interacting taxa in the ecosystem, and observing how the two groups populated such structures. In particular, the HC group showed an even population of modules, while CRC showed a strong dominance of one module over the others. This module was particularly overabundant with species previously reported as potential opportunistic pathogens or CRC-associated species (e.g., *Escherichia coli, Akkermansia muciniphila, Alistipes* spp*., Bacteroides* spp*.*)^[73,74]^. The interpretation of the overabundance network, particularly when there is robust segregation of nodes into modules, aids in identifying potential sub-assemblies of microorganisms within the microbial community. Their identification may contribute to the understanding of the underlying factors that explain the observed conditions. In our case, the canonical statistical analysis of the relative abundance did not allow for detecting differences in the species populating module 3 in the global network; nevertheless, the interpretation is in agreement with the previous results in the literature, which makes the implementation of this analysis potentially very informative.

## CONCLUSIONS

In recent years, networking approaches are gaining more and more attention in the microbiome field, and tailored tools are continuously being developed to address issues related to the nature of microbiome data. Aside from standard statistical methods and discriminant analyses, networking allows inferring deeper relationships between the microorganisms of the microbial community, possibly providing additional insights into the forces that shape the ecosystem. Far from being the gold standard technique for microbial analyses, networking analysis is indeed a great tool for microbiome studies, especially for describing a novel ecosystem as well as for deriving further (compositional and/or functional) insights from well-characterized ecosystems such as human feces. The strength of networking is that the analyses can be conducted at multiple levels, for example, on a local or global scale, and considering topological (modularity, centrality, hub) or ecological (cohesion, keystone) parameters in order to produce an all-round assessment of the community structure.

On the other hand, the limitations of networking analysis applied to microbiome data are yet considerable, given the need for large sample sizes, computational power, and the struggle to implement statistical tests on the final outputs, as most of the time, they consist of single values. Nonetheless, the added boost to the ever-growing microbiome field provided by networking techniques should be acknowledged and implemented. With the development of increasingly tailored tools and - perhaps in the future - machine learning methods to help identify patterns and meaningful relationships between nodes, networking might actually become the gold standard for microbiome analysis.

### Box 1. Important terms related to networking analysis.

**Node -** A node is a fundamental unit within the network representing an individual entity; in a microbiomea network analysis, a node is represented by a bacterial taxon at a specific level (e.g., genus, species, etc.).

**Edge** - In a network analysis, an edge is basically the graphical representation of an interaction between nodes, which can be positive or negative, weighted and directed or not. In a microbiome network, edges are typically just positive or negative and weighted, showing a correlation in terms of abundance.

**Module** - Module refers to a collection of nodes that are closely interconnected with each other, and with relatively fewer connections to nodes outside the group.

#### Node characteristics that define the node centrality in a network.

**Degree -** This refers to the count of edges that connect a chosen node with the rest of the nodes in the network.

**Betweenness centrality** - It measures the extent to which a vertex lies on paths between other vertices/nodes.

**Closeness centrality** - Reciprocal of distance sums from a specific node to all reachable nodes.

**Distance** – The total weight of all edges within the shortest path between two nodes.

**Hub node** - A node that has more connectivity within the network than other nodes, based on the node centrality values.

**Keystone node** - A node crucial for the observed network structure, meaning that removing this node from the network alters significatively its layout. Not all hub nodes are keystones and *vice versa*, as the definition of the two differs significantly. Brute force leave-one-out approaches can be used to detect this type of node^[40,75]^.

## DECLARATIONS

### Availability of data and materials

All the sequences used for the case study are publicly available and the accession list can be found in Supplementary Data 1.

### Copyright

© The Author(s) 2023.
