# Supplementary material for: Connect the dots: sketching out microbiome interactions through networking approaches
Source: Microbiome Res Rep. 2023 Jul 18;2(4):25. doi: 10.20517/mrr.2023.25 (PMC10696587; doi:10.20517/mrr.2023.25)

Supplementary Figure 1

Supplementary Figure 1. Bacterial species differentially represented in the gut microbiome of patients with colorectal cancer versus healthy controls in the case study. Boxplots showing the relative abundance distribution of species differentially represented between healthy controls (HC) and colorectal cancer patients (CRC), as determined by Wilcoxon rank sum tests followed by False Discovery Rate error correction. *: FDR < 0.05; **: FDR < 0.01; ****: FDR < 0.0001. CRC: Colorectal cancer patients; HC: healthy controls; FDR: false discovery rate.

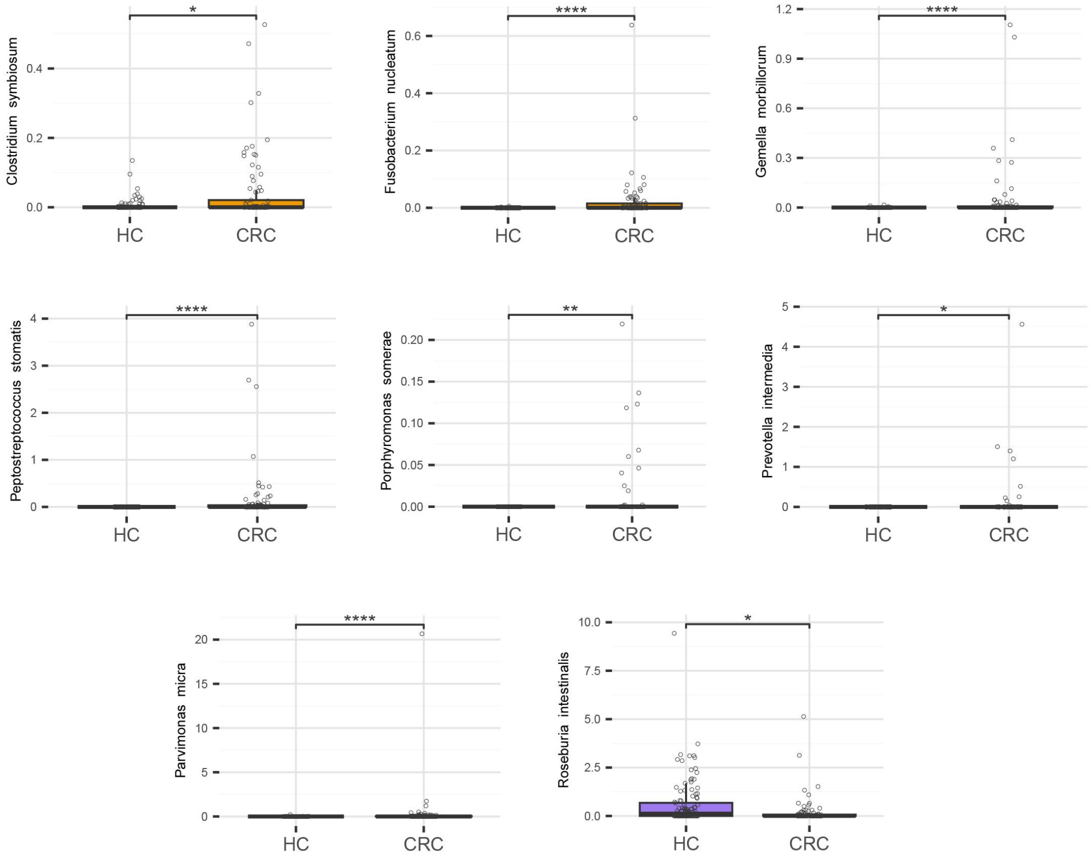

Supplement: Supplementary file 2 [file mrr-2-4-25-SupplementaryMaterials.pdf]
